# The challenge of cognitive science for medical diagnosis

**DOI:** 10.1186/s41235-022-00460-z

**Published:** 2023-02-09

**Authors:** Pat Croskerry, Samuel G. Campbell, David A. Petrie

**Affiliations:** Department of Emergency Medicine, Faculty of Medicine, Dalhousie University, Halifax, Canada

## Abstract

The historical tendency to view medicine as both an art and a science may have contributed to a disinclination among clinicians towards cognitive science. In particular, this has had an impact on the approach towards the diagnostic process which is a barometer of clinical decision-making behaviour and is increasingly seen as a yardstick of clinician calibration and performance. The process itself is more complicated and complex than was previously imagined, with multiple variables that are difficult to predict, are interactive, and show nonlinearity. They appear to characterise a complex adaptive system. Many aspects of the diagnostic process, including the psychophysics of signal detection and discrimination, ergonomics, probability theory, decision analysis, factor analysis, causal analysis and more recent developments in judgement and decision-making (JDM), especially including the domain of heuristics and cognitive and affective biases, appear fundamental to a good understanding of it. A preliminary analysis of factors such as manifestness of illness and others that may impede clinicians’ awareness and understanding of these issues is proposed here. It seems essential that medical trainees be explicitly and systematically exposed to specific areas of cognitive science during the undergraduate curriculum, and learn to incorporate them into clinical reasoning and decision-making. Importantly, this understanding is needed for the development of cognitive bias mitigation and improved calibration of JDM in clinical practice.

## Introduction

In the early part of the sixteenth century, the Swiss physician von Hohenheim, credited with being the originator of clinical diagnosis in medicine, had attracted the appellation Paracelsus (exalted above or beyond Celsus, the first-century Roman medical authoritarian). Among other notable innovations, he famously pronounced that Medicine was both a science and an art dealing as it did ‘with the very processes of life, which must be understood before they may be guided’ (https://en. wikipedia.org/wiki/Paracelsus accessed 12 Jan 2022). It may be argued that this claim to being an art appears to have excused Medicine from becoming a stand-alone science, or at least blurred the edges of what a medical science might look like—probably not quite what Paracelsus had in mind as much of his work was aimed at dispelling myths and superstitions, and putting Medicine on firmer ground.

Through the Age of Enlightenment, while other basic sciences were establishing evidence-based footings, Medicine was less so. Even as late as the mid-twentieth century, clear warnings that formal (actuarial, statistical) methods would reliably outperform clinical (subjective, informal) methods of prediction, appeared to go unheeded (Meehl, 1954). It wasn’t until the 1980s that ‘evidence-based medicine’ proper began to emerge with the work of David Eddy (Eddy, 1982) and others (Evidence-Based Medicine Working Group, 1992) (for comparison, imagine in the late twentieth century, discussing the merits of evidence-based physics, or evidence-based chemistry). There appears to have been, and continues, a significant lag time in Medicine’s willingness to take its place as a fully fledged science, at least as far as cognitive science is concerned. Admittedly, many features of clinical medicine generate inherent uncertainties that are often difficult to resolve, not the least of which is the variability in the course that many diseases take, as well as the multiple ways in which they may be expressed and interpreted by those who have them. Further, the practice of medicine has some inherent non-scientific aspects without which it would be markedly less effective overall. Sympathy, compassion, empathy, justice, forbearance, non-attributional judgement, kindness, and a variety of other qualities would be considered within the ‘art’ of medicine, all of which may be significant in the healing process. Nevertheless, the ‘art’ moiety appears to have allowed some foot-dragging and bending of the rules, such that physicians could (and still do) act on nebulous and unexamined hunches, gut feelings, intuitions, and impressions about medical problems, even though significant morbidity and mortality for their patients might ultimately be at stake. This is not to say that a well-informed hunch may be less reliable than an analytic decision, just that an awareness of the cognitive science behind the two is important.

When the consequences associated with medical errors are taken into account, the complexity and range of processes involved in Medicine intrinsically appear more challenging than the domain of pure science. This, perhaps, provides some excuse for a want of scientific rigour in some areas. Notably, there has been a distinct hesitation in the uptake of cognitive science into clinical decision-making and reasoning.

### Medical diagnosis

After hearing the presenting complaint from the patient or from a collateral source (family/friend/caregiver), the assessment typically involves taking various aspects of the patient’s history (history of the present illness, past medical history, family history and psychosocial history), review of systems, physical exam and diagnostic tests. Usually, this part of the assessment is focused on the patient’s complaint and symptoms, deciding which questions and/or tests are relevant to the case. Those selected are posed to rule in a possible diagnosis and rule out other diagnoses that could have caused the presenting complaint. This part of the assessment ends when the clinician feels they have enough information to identify the diagnosis. A common cause of diagnostic error is “premature closure” when the clinician makes a premature diagnostic decision without gathering sufficient information to rule out other potential causes of the presenting complaint. This may be due to an unpacking failure, i.e. a failure to elicit sufficient information to include the range of diagnostic possibilities in a case. The clinical reasoning process is not simply deciding which is the most likely diagnosis given the information available, it includes deciding what information to collect that will help to determine the diagnosis. Every clinician understands this, but non-clinicians may not recognize the reasoning process involved in carrying out a patient assessment.

It is evident that a number of decisions have to be made along the way in the process of making a medical diagnosis. They range from simple to complex. Thus, the science of decision-making becomes very important and relevant to the accuracy of the diagnostic process. However, the importance and relative value of medical reasoning and decision-making does not appear to have received adequate recognition.

In 2005, Eddy remarked that in the 1970s ‘Medical decision-making as a field worthy of study did not exist’ (Eddy, 2005), and in a comprehensive review of clinical reasoning in 2011, Pelaccia et al. (2011) noted:

“We must recognize that the academic environment of medical students hardly promotes the active development of clinical reasoning. Indeed, although medical educators share the view of clinical reasoning as a major determinant of physicians’ expertise, it is not often an explicit educational objective in medical universities. Thus, the development of clinical reasoning abilities will most often remain secondary in comparison with knowledge and practical skills acquisition. Many believe that this competence will be gained gradually and naturally over time, through clinical experience. Other teachers may believe that reasoning relies on personality traits that will hardly be gained during medical studies if they are not already present at entry to medical school.”

Yet, how clinicians think, and in particular how they think about clinical reasoning and diagnosis is, arguably, their most important skill. As Nuland noted: “It is every doctor’s measure of his own abilities; it is the most important ingredient in his professional self-image,” (Nuland, 1994) and they would be the first to admit it. Even so, the development of a scientific (cognitive) understanding of clinical decision-making has remained a significant challenge.

With the emergence of the cognitive revolution in the mid-twentieth century, and the application of the scientific method to the study of human cognition, significant efforts were made to develop insights into clinical reasoning and how to teach it. In 1979, a team of psychologists authored one of the first specific treatments of the topic with *Medical Problem Solving: An analysis of clinical reasoning.* Interestingly, their approach traced its origins not to behaviourism, the dominant school of thought in North American Psychology at the time, but to ‘European thought psychologists and Gestaltists’ (Elstein et al., 1979). An equally influential book followed by the clinicians Kassirer and Kopelman: *Learning clinical reasoning *(Kassirer & Kopelman, 1991), for the first time in a medical text, had an explicit focus on the influence of cognitive bias in clinical decision-making. Both used the technique of directly observing experienced clinicians thinking aloud while evaluating medical problems in clinical cases, either simulated or narrated. Variously, comprehensive reviews have examined a number of methodological challenges in this area of research (Audétat et al., 2013; Elstein et al., 1990; Pelaccia et al., 2011), but progress, overall, has been relatively slow.

In a 2017 survey of clerkship directors of Internal Medicine from 95 medical schools in the USA, training sessions dedicated to key clinical reasoning topics did not exist in 57% of programmes. Lack of curricular time and faculty expertise were reported as the main barriers to teaching these concepts (Rencic et al., 2017). The focus in Medicine generally lies on other things: understanding disease at a cellular level, and developing improved technology, testing, and imaging of it. But throughout, less emphasis is placed on how to think and reason rationally about the diagnosis of disease. Thus, the failure to explicitly teach clinical decision-making in the majority of US medical schools means that the significant gains that have been made in the cognitive science of decision-making over the last 50 years have been barely acknowledged. In particular, until recently there was little awareness of what is now accepted as the basic platform of human decision-making, dual process theory, which describes two types of decision-making: System 1 encompasses a wide range of fast, non-verbal, processes based on pattern recognition known as intuitive decision-making, while System 2 processes are slower, verbal, deliberate, less prone to error and known as analytical decision-making. This approach originated with the work of Schneider and Shiffrin in 1977 (Schneider & Shiffrin, 1977), although the dichotomous nature of decision-making appears to have been well appreciated by Thomas Paine much earlier in 1794 (Paine, 2018).

“Any person, who has made observations on the state and progress of the human mind, by observing his own, cannot but have observed, that there are two distinct classes of what are called Thoughts; those that we produce in ourselves by reflection and the act of thinking, and those that bolt into the mind of their own accord. I have made it a rule to treat those voluntary visitors with civility, taking care to examine, as well as I was able if they were worth entertaining, and it is from them that I have acquired almost all the knowledge that I have.”

The model, originally described as a ‘two-process theory of human information processing’ (Schneider & Shiffrin, 1977), has been adapted for medicine through a succession of iterations. Elstein conceived of it as a distinction between *clinical* prediction which was ‘artful and qualitative’ and *actuarial* or *statistical* prediction which was ‘formal and quantitative’ (Elstein et al., 1979). Drawing on an extensive body of earlier work by Hammond and others (Hammond & Sage, 1990), Dawson listed characteristics of the two systems referring to them, respectively, as intuitive and analytical (Dawson, 1993). More recently, they have been widely known as System 1 and System 2, popularised by Kahneman (2011) or as Type 1 and Type 2 processes preferred by Evans (Evans, 2008), and by the Stanovich group, prominent in this area of research (Stanovich et al., 2014). A basic model of the two systems in medicine summarising the intra-operative characteristics of the two systems was later proposed (Croskerry, 2009). The model is now in widespread use throughout the various disciplines of clinical medicine.

The most important decisions that physicians make are about the patient’s diagnosis, a process that is both complex and complicated, and which qualifies as a complex adaptive system (CAS) within the multiple CASs of healthcare. Multiple independent and interdependent variables that impact the clinical reasoning process have been described (Croskerry, 2018), emphasising that the cognition that underlies diagnostic reasoning occurs not in isolation but in situation or context, with interactions between multiple variables (Norman, 1993). Thus, there is a complex interplay of clinician, patient, practice setting and environmental variables in the diagnostic reasoning process. Situated action or situativity theory includes situated cognition, situated learning, ecological psychology, and distributed cognition (Durning & Artino, 2011; Merkebu et al., 2020).

The variables are segregated into six clusters: *A:* demographics and other characteristics of the decision-maker; *B*: cognitive characteristics of the decision-maker; *C*: homeostatic and other challenges to the decision-maker; *D*. ergonomic and other characteristics of the decision-maker’s environment; *E*: characteristics of the disease itself and the way in which it presents; and F: characteristics of the patient, their culture and family, and the influence of other patients. There are probably more, as well as the additional potential for many significant second- and higher-order interactions between the independent variables, combining to influence diagnostic accuracy. Consider, too, that some variables listed, such as personality in Cluster B, encapsulate a subset of other variables (e.g. cognitive empathy, emotional intelligence, conscientiousness and others) which complicates things further. Also, many of these variables are nonlinear, for example, clinician factors such as age and experience change over time, as well as vulnerability to certain JDM biases (Pitz & Sachs, 1984) with ageing (Strough et al., 2011). Similarly, Cluster D factors would be expected to change as system design improves over time, as would Cluster E given that knowledge about specific diseases continues to improve over time. Such complexity creates a significant potential for error.

Approximately 75% of diagnostic failures appear to have a cognitive origin (Graber et al., 2005), a finding that has been strongly endorsed recently in a major review from the US Agency for Healthcare Research and Quality (AHRQ) (Newman-Toker et al., 2022), which looked at diagnostic failure in Emergency Departments (ED). Out of 130 million ED visits annually, over 7 million diagnostic errors were estimated, of which 350,000 were associated with serious harm, including 250,000 deaths. Almost 90% of diagnostic error malpractice claims involved clinical decision-making or judgement failures. Other cognitive sources of error noted were inadequate clinical knowledge, skills, and reasoning, especially in atypical cases. In other studies, conceptual knowledge deficits have not been found to be a significant source of error (Croskerry, 2020a; Graber et al., 2005; Gruver & Freis, 1957; Kiesewetter et al., 2016); clinical judgement and reasoning therefore appear to be the primary source of cognitive failure.

Even when the patient is admitted to the hospital, the morbidity and mortality associated with diagnostic failure are significant. Leape, Berwick, and Bates, all luminaries in the field of patient safety, estimated an annual mortality rate in the range of 40,000–80,000 (Leape et al., 2020). When outpatient settings, other than the ED are taken into account (primary care, walk-in clinics), where the majority of patients are seen, the number affected by diagnostic error would be orders of magnitude higher. The estimated diagnostic failure rate across the board in medicine is 10–15% (Berner & Graber, 2008; Elstein, 1995), depending on which medical discipline is involved. There is considerable variability: in the visual specialities (radiology, dermatology and anatomic pathology) the reported rates are relatively low, probably less than 5%, whereas in Psychiatry the rate is much higher, around 45% (Silveira & Rockman, 2021). The higher estimates for Psychiatry appear due to a variety of factors which are discussed further below.

### Pattern recognition

**Fig. 1:**
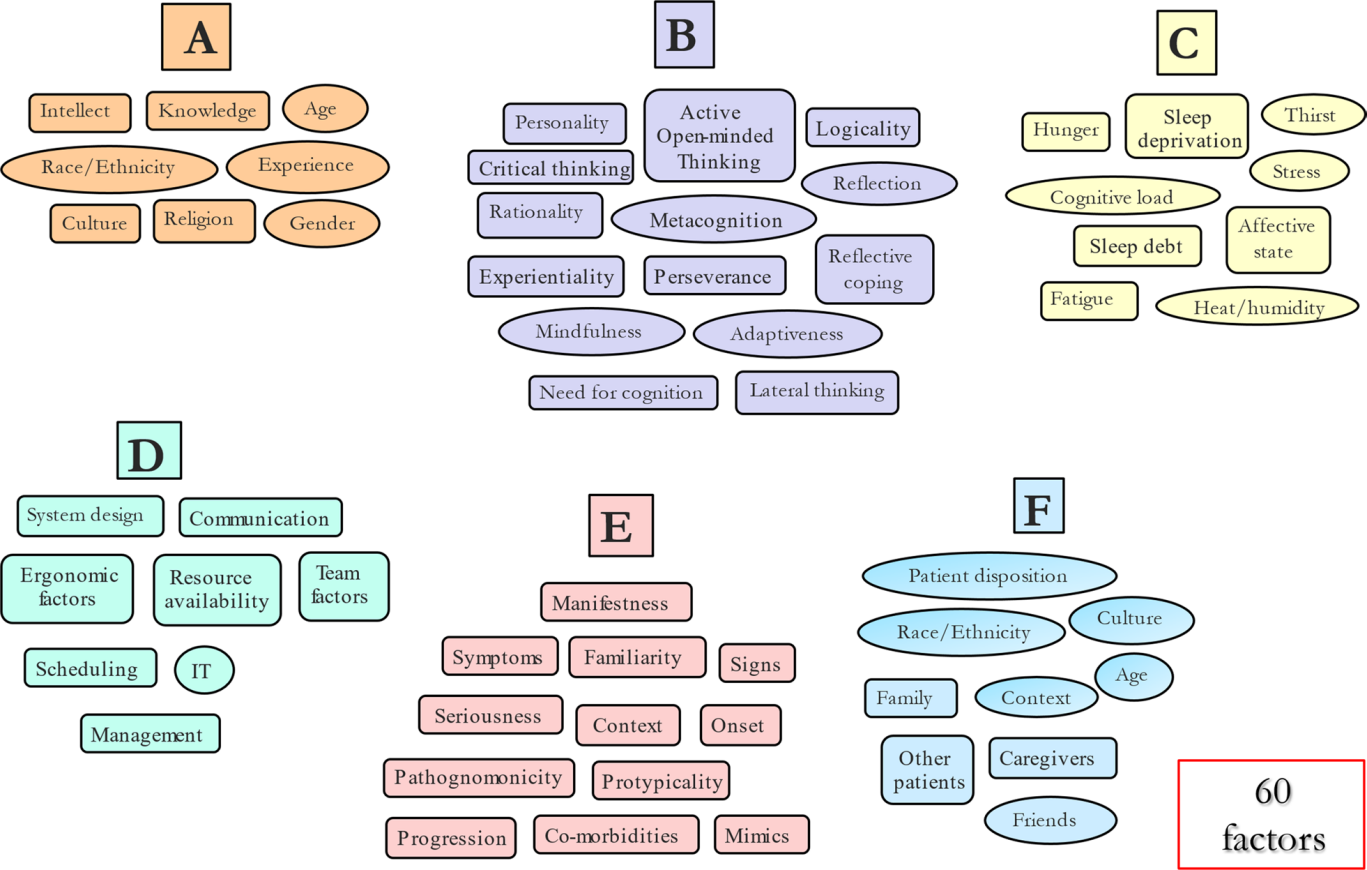
Six clusters of factors that may impact the diagnostic process. Adapted from Croskerry (2018) See text for details

**Fig. 2:**
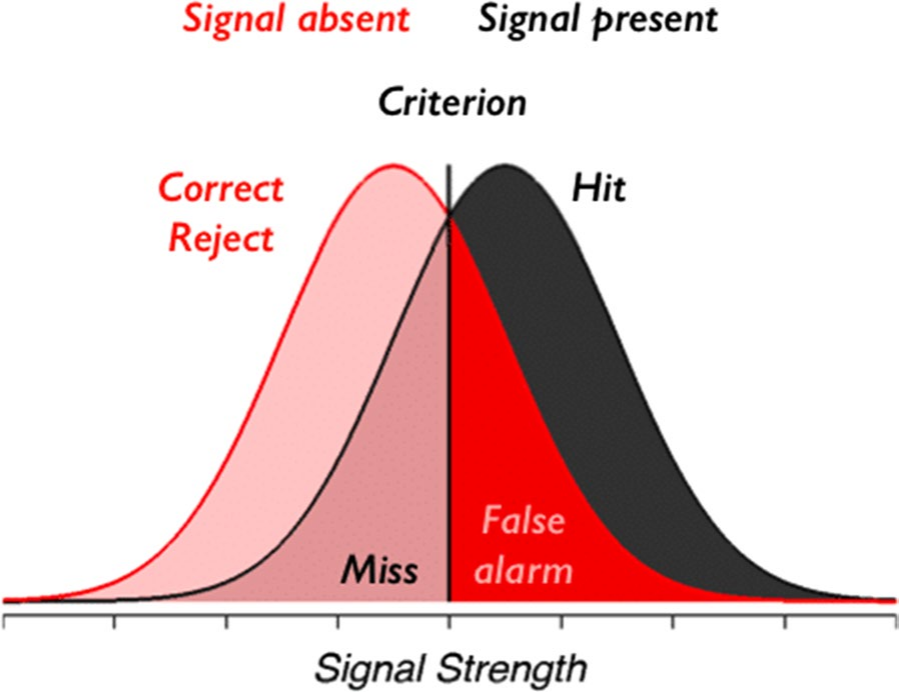
Signal detection

The assignment of a medical diagnosis begins with a pattern recognition systematizing process based on symptoms the patient may be experiencing and relating, signs they may be showing, and/or information from other sources. Systematizing of patterns is seen as the most basic and essential of human skills. It is a drive towards analysing and understanding systems that are governed by rules which operate according to logic and scientific laws. Essentially, systematizing allows us to predict how systems are most likely to behave (Baron-Cohen, 2009). Thus, pattern-seeking is a starting point for systematizing and understanding ill health. Cluster E factors (Fig. 1) deal with the characteristics of the disease being diagnosed. Of paramount importance is the pattern that the disease presents, and the ability of the clinician to detect and distinguish a meaningful signal from the noise with which it is typically associated. This problem was identified in some of the earlier work on psychophysics and signal detection theory in psychology (Green & Swets, 1966) (Fig. 2). Through detection, discrimination and identification, the physical properties of stimuli are translated into signals that have intrinsic meaning based upon the knowledge base that has been established in the usual course of medical training. This, in turn, allows for a meaningful response to be made, followed by a decision about how the disease will be managed. Where the consequences of missing a crucial signal are high, the criterion may be moved to the left, which would include more false positives.

**Fig. 3:**
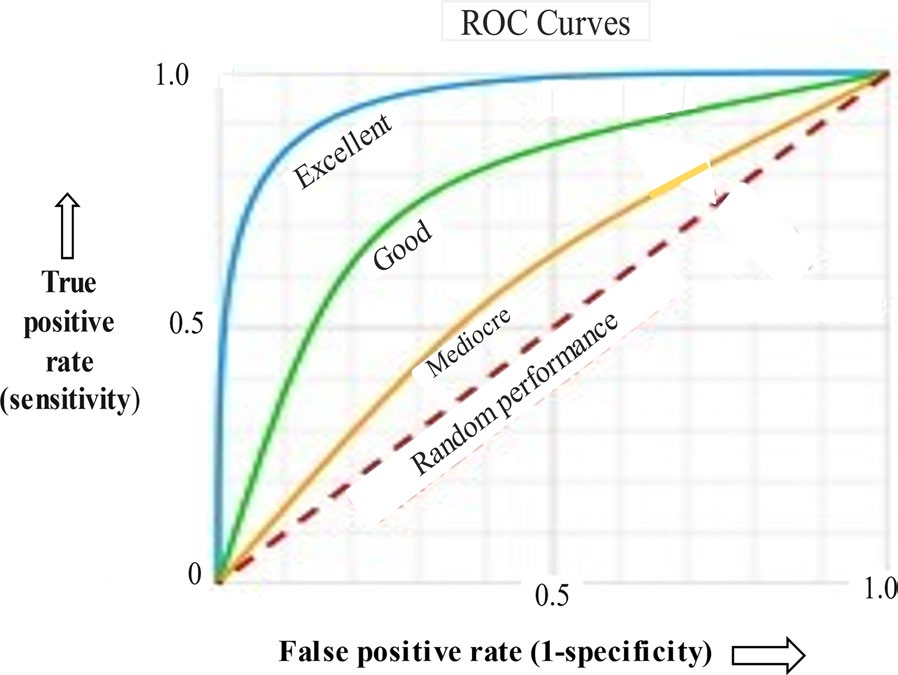
Receiver operating curves (ROC) for different levels of noise. See text for details

A major feature of the diagnostic process is that when there is less uncertainty and minimal noise associated with a signal, the more likely it is to be detected and accurately identified. Thus, in the visual specialties (dermatology, radiology, anatomic pathology) where noise is minimal, receiver operating characteristics (ROC) will show good calibration with high true positive and low false positive rates of diagnosis, with an overall accuracy of 1–2% (blue curve in Fig. 3).

In the intensive care unit (ICU), resources are usually excellent with high physician-to-patient and nurse-to-patient ratios, as well as ready availability of sophisticated technical equipment. Nevertheless, a systemic analysis of 31 different studies in the medical literature from 1966 to 2011 involving autopsy-confirmed diagnostic errors found a diagnostic failure rate that was still surprisingly high. About 1 in 4 cases had a missed diagnosis (28%), which in 8% of cases may have caused or contributed to the death (Winters et al., 2012). Part of the explanation may be that multisystem disorders, typically suffered by critically ill patients, may result in one system’s dysfunction/disease being hidden behind another. Interestingly, common diseases were most often missed, partly because they are simply more common, but also because the main focus of attention was on more complex diseases.

In internal medicine, emergency medicine and family practice, where the overwhelming majority of patients are seen, the misdiagnosis rate is about 10–15% (Berner & Graber, 2008), corresponding to the green curve. Diagnostic performance in Psychiatry, the least well-calibrated of all the disciplines, corresponds to the yellow curve. Unlike other disciplines, Psychiatry is unique in that, presently, it lacks reliable biomarkers, imaging technology, or other objective measurements that can identify and objectify psychiatric disease. The nature of psychiatric illnesses, characterised as it is by complexity, nonlinearity and unpredictability (Silveira & Rockman, 2021), is also very challenging. The psychiatric diagnosis essentially rests on descriptions of behaviour and how they correspond to diagnostic entities defined by a consensus of experts, published in the Diagnostic and Statistical Manual of Mental Disorders, now in its 5^th^ iteration (American Psychiatric Association, 2013). In terms of the objective refinement of diagnosis, Psychiatry appears to be where Internal Medicine was many decades ago.

### Disease manifestness

**Fig. 4:**
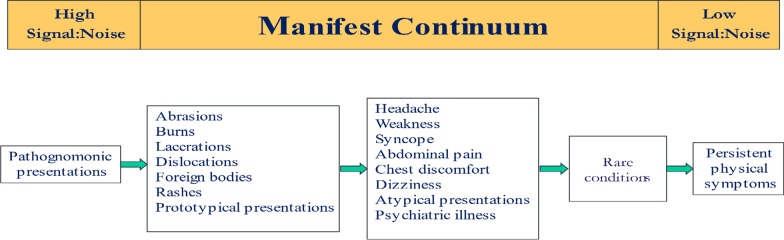
Manifest continuum of diseases. Adapted from Croskerry (2020) See text for details

Pattern recognition critically depends on the manifestness of the signal which depends, in turn, on the level of associated noise. The less noisy, the more distinct its features, the greater degree of manifestness, and the more reliably it will be detected (Croskerry, 2020) (Fig. 4). Highly manifest diseases are referred to as pathognomonic. For example, the rash of herpes zoster has explicit features that few other rashes have: clusters of vesicles, patches of erythema, involving one or two dermatomes, stops at the midline, and is extremely painful; its signal-to-noise ratio is very high. Moving along the continuum, common injuries and prototypical presentations are also relatively low in noise and usually straightforward to diagnose. In the next category, the noise level is considerably higher. Chest discomfort has about 25 possibilities, while headache has about 300. Atypical presentations of the disease include common diseases which may be missing common signs. In acute coronary syndromes, for example, chest pain may be absent in 1/3 cases. Atypical presentations are a major source of diagnostic failure (Newman-Toker et al., 2022). At the next level are rare conditions. Their features may be quite manifest but they are extremely uncommon, for example, a cerebrovascular accident (CVA) or stroke in a child. The noise here arises from the mismatch between the age of the patient and the disease. It may not be considered on the differential diagnosis list not because its manifestation is low but because in that age group it is virtually unknown (2 in every 100,000 patients). The degree of manifestness depends, to some extent, on context, and in this case, age is an important contextual factor. In elderly patients, classic symptoms, such as a fever, may be blunted or missing altogether. Finally, at the extreme of the continuum lie a group of ill-defined diseases that are difficult to diagnose such as fibromyalgia, irritable bowel syndrome, multiple chemical sensitivities and others, and may include entities that essentially defy diagnosis, known as medically unexplained symptoms (MUS) or diseases, or as patients with persistent physical symptoms (PPS) (Marks & Hunter, 2015).

### Establishing cause

At its most basic, the process of making a diagnosis is an effort to establish a cause for the patient’s symptoms, essentially: *if P (symptoms) then Q (disease)*. Patients themselves commonly attribute causation through self-diagnosis, often aided by searching online for a disease that matches their symptoms, or, in the especially vulnerable, symptom searching for diseases they might have, referred to as cyberchondriasis. Medical students are similarly vulnerable. A humorous account by the medical student, George, is given in Jerome’s classic Three Men in a Boat (To Say Nothing of the Dog) (Jerome, 1889), published in 1889:

“I remember going to the British Museum one day to read up on the treatment for some slight ailment of which I had a touch—hay fever, I fancy it was. I got down the book, and read all I came to read; and then, in an unthinking moment, I idly turned the leaves, and began to indolently study diseases, generally. I forget which was the first distemper I plunged into—some fearful, devastating scourge, I know—and, before I had glanced half down the list of “premonitory symptoms,” it was borne in upon me that I had fairly got it. I sat for a while, frozen with horror; and then, in the listlessness of despair, I again turned over the pages. I came to typhoid fever—read the symptoms—discovered that I had typhoid fever, must have had it for months without knowing it—wondered what else I had got; turned up St. Vitus’ Dance—found, as I expected, that I had that too,—began to get interested in my case, and determined to sift it to the bottom, and so started alphabetically—read up ague, and learnt that I was sickening for it, and that the acute stage would commence in about another fortnight.”

Not surprisingly, causation is often confounded by correlation. Two factors that are highly correlated might appear to be causally related, but are not necessarily. A fundamental problem with establishing a causal relationship between symptoms and disease is that a one-to-one matching of disease to symptoms is not possible. There aren’t enough symptoms to go around for the estimated 19,000 diseases recognised by the International Classification of Diseases (ICD) in its current revision (World Health Organization, 2018). The human body can only express itself in so many ways, probably with fewer than 200 symptoms, so diseases outnumber symptoms about 100:1. Combinations of symptoms increase the possibility of distinguishing one disease from another, but may also obfuscate the classification of disease. For example, the Diagnostic and Statistical Manual of Mental Disorders (DSM–5) now allows 636,120 combinations of symptoms that qualify for the diagnosis of Post-Traumatic Stress Disorder (Galatzer-Levy & Bryant, 2013), currently ranked the fourth most commonly described psychiatric disorder.

Nevertheless, in practical terms, there are still not enough symptoms to go around. From a logical standpoint, *if P then Q* rarely exists except for pathognomonic conditions, then modus ponens translates into *if P then potentially many Qs*. In the case of the symptom of headache, as noted, *P* may mean 300 options for Q. Similar challenges exist for laboratory data, e.g. a D-Dimer level (referring to a protein fragment that is made when a blood clot dissolves) may be elevated by several benign causes, but importantly by both blood clots in the lungs, and a dissection of a major artery, two quite different diagnoses and both potentially fatal conditions. The process of establishing reasonable causation often requires gathering multiple ancillary data in the confirmation and disconfirmation process of reaching a diagnosis. When the process fails, it may be due to unpacking failure (a failure to unpack sufficient data in the initial assessment) (Redelmeier et al., 1995) which in a series of clinical cases of diagnostic failure from emergency medicine ranked fifth overall in frequency (Croskerry, 2020a). Unpacking failure is a failure to elicit any relevant information in a patient’s history which might facilitate consideration of a more complete range of diagnostic possibilities. The relatively common confirmation bias arises through a predilection for seeking confirmation over disconfirmation.

### Proximal and distal

In an effort to understand the causes of diagnostic failure, various failings in the processes that have been used in medical diagnosis have been targeted, e.g. deficiencies in reviewing a patient’s history, in conducting a clinical exam, in test ordering and interpretation, and others. In the Diagnostic Error Evaluation and Research (DEER) taxonomy, while cognitive factors are acknowledged as contributory (Schiff et al., 2005), the clear emphasis is on tangible measurable aspects of the diagnostic process. Invisible cognitive aspects receive little attention. This taxonomy has been widely adopted in studies of diagnostic failure, and while helpful as a first step in the analysis of diagnostic failures, it inevitably puts the emphasis on proximal explanations, rather than getting to the distal causes where the problem actually lies along the chain of causation (Croskerry, 2016). For example, in the case review of a diagnostic error, a ‘failure to elicit a critical piece of history’ in the DEER classification may be the problem identified for a diagnostic failure, whereas a more helpful analysis might identify a particular JDM bias (anchoring and adjustment, availability and others) that led to an unpacking failure and an incomplete history, as the distal explanation for the error in the first place. By ‘distal’ here we mean closer to the actual origin of the error. However, in order to more reliably identify distal antecedents of an error, a cognitive autopsy is required (Croskerry, 2003; Croskerry et al., 2008a), referring to a root cause analysis of a medical adverse event. Presently, a conventional root cause analysis typically pursues an iterative interrogation to investigating the root cause of a problem, often by repeatedly asking “Why”, to get to the distal factors where the problem may have originated. It relies very much on visible, tangible explanations, and does not usually allow for assessment of how individuals think. In contrast, a cognitive autopsy explicitly attempts to identify cognitive factors that underlie decisions that were made, and, when successful, moves the explanation more distally along the chain of causation (Croskerry & Campbell, 2021).

Invariably, attempts to understand distal causes of error by conducting a cognitive autopsy are less tangible, more challenging and time-consuming. Further they require someone trained in the process, able to do cognitive interviewing (Geiselman et al., 1984), and with a good knowledge of JDM biases and other decision-making failures. The findings from the autopsy will only be meaningful to those who understand the JDM process and have a non-attributional approach towards identifying cognitive failures. This requires a higher level of awareness of the concepts of JDM among learners and managers than currently exists, already identified as a major impediment in teaching clinical decision-making (Rencic et al., 2017).

### Heuristics and biases

Although familiar to cognitive scientists, most medically trained people have a limited understanding of what is meant by these terms. Various explanations offered tend to oversimplify things, suggesting that heuristics are simply mental short cuts, with biases resulting when they go wrong. Part of the problem has arisen over how the term ‘heuristic’ has been used (Croskerry et al., 2017a). Some have used it as a tool or strategy, especially in situations of uncertainty where all possible options, and their consequences, are not known to the decision-maker (Artinger et al., 2014) for example, ‘heuristic’ can be used to describe a general strategic concept or rule that might improve decision-making in chess, e.g. *control of the center*, where the player deliberately chooses an approach that might prove beneficial overall. At other times, heuristics may be no more than simple associations that might occur such as in the JDM bias anchoring and adjustment where the decision-maker may unconsciously lock onto a salient feature of a patient’s presentation at the outset and later fails to make any adjustment; in the process, no deliberate choice is made. To clinicians, the notion of deliberately choosing to cut corners may sound precarious in the context of patient health and safety, especially when it is connected with the negative term ‘bias’, and may have exacerbated aversion to the word. To the cognitive scientist, instead, Type 1 processing is seen as autonomous, reflexive, largely unconscious and non-deliberate, so the decision-maker does not actively choose to use it, and therefore is not guilty or culpable in an immediate sense. Accountability may follow later, however, if a decision-maker is aware of the effects of JDR biases and does not take meliorative action in the longer term (Stark & Fins, 2014).

Current definitions of ‘thinking’ typically imply it is a deliberate act, so there should not be any actual ‘thinking’ in Type 1 processing yet we often hear clinicians refer to ‘thinking’ in System 1 being influenced by heuristics, even though there may be no deliberate choosing of a heuristic strategy where a JDM bias is involved. It often seems in clinical medicine that the heuristic may be little more than a simple association between stimulus pattern and response. Such associations may trigger anchoring, which, if no judicious adjustment follows, may lead to premature closure in the problem-solving process. These two cognitive biases, anchoring and adjustment and premature closure, are common in clinical medicine.

There is an additional problem in the use of the term ‘bias’. For the non-cognitive scientist, including most medically trained clinicians, the word “bias” connotes negative features, such as weakness of judgement, lack of objectivity, and vulnerability to invisible forces. To the psychologist, however, bias is an aspect of behaviour worthy of study, a unique feature of human cognition, and some social/sociological biases aside, it is not necessarily a failing. As noted, however, if we have been made aware of the negative outcomes associated with JDM biases and have failed to attempt to correct them, it then becomes an ethical issue (Stark & Fins, 2014).

Importantly, JDM biases provide consistent explanations for why clinicians do the things they do (Croskerry & Campbell, 2021). Deutsch, the Oxford physicist, has argued that explanation is the bedrock of reason and, historically, many theories have been rejected because they contain bad explanations, not because they actually fail experimental verification. For the diagnostic process, it has been argued that, unless a good explanation is provided for why it fails, it is likely that the same errors will continue to be made. The objective exposure of biases often provides coherent, distal explanations for why events occurred.

### Cognitive bias mitigation (CBM)

There appear to be a variety of reasons to explain clinician reluctance towards accepting cognitive science as an essential component of medical JDM. The major ones we have identified are described in Table 1. One of them, in particular, appears to present a stumbling block, that bias is difficult to correct so why invest time on it? Certainly, soon after Kahneman’s and Tversky experimental findings were published in the 1970s, reports on attempts to fix biases appeared. Fischoff et al. (1982) and others (Kahneman, 2011; Wilson & Brekke, 1994) generally signalled gloom and doom, a mood that has prevailed for a number of years.

**Table 1:** Factors that may impede medicine’s acceptance of the integral role played by JDM biases in medical decision-making

Factor	Comment
Lack of cognitive science training in medicine	Medicine has a history of insularity and going its own way. Cognitive science may suffer from the not-invented-here (NIH) syndrome
Difficulty in incorporating cognitive science into the curriculum	Medical undergraduate curricula are usually full and there is little room for additional material, so traditional content and the status quo prevails. With constant updates of existing content, it is difficult to introduce new material, especially any that does not appear to have immediate clinical relevance
Lack of appropriately trained personnel to teach about cognition	Appropriate instructors in cognitive science could be recruited from other disciplines, once Medicine recognises this need; later, it can develop its own
Invisibility of cognitive processes	Historically, Medicine has emphasised the tangible and visible. Sophisticated imaging allows clinicians to see the problem even down to a cellular level. However, the imaging of cognitive processes is presently a formidable challenge
Discomfort and even antipathy against the term ‘Heuristics and Biases’	‘Heuristics’ is an uncommon term in medicine and not usually used in clinical decision-making. Further, the use of the word ‘bias’ tends to get seen negatively rather than as an objective way of looking at cognitive behaviour. Most clinicians would not like to hear their decisions described as biased. Describing the problem as a JDM bias may displace the blame somewhat and make it less personal
Status quo and extra work	Many physicians are busy and typically overworked. Learning about cognitive science is extra work added to already busy schedules. Cognitive miserliness is likely with a tendency to preserve the status quo
Denial, discounting and distancing	Physicians hold dear their ability to accurately diagnose illness. Confronted with the failure rates reported they may engage defensively in alternate explanations rather than accept an understanding of cognitive science as a necessity
Cognitive science is a novel concept to many	While clinicians readily accept the need for regular updating of their existing knowledge, they may resist learning about a new approach not traditionally covered in the medical curriculum
Bias is difficult to correct, so why invest time in it?	CBM is challenging and may require significant effort, but recent work suggests that some CBM strategies may be effective
Complexity of the diagnostic process	Overall, the medical diagnostic process is both complicated and complex. With a limited understanding of the process itself, many clinicians would be reluctant to add a new approach to their current understanding of it

It should have come as no surprise, in fact, that bias is difficult to overcome. The response tendencies that underlie bias are established in a variety of ways and laid down on a seemingly deep and powerful base. They might have an innate origin, surviving as proof of effective decision-making from our ancestral past, they might be established through repeated explicit learning and over-learning, others could be associated with our emotions, positive or negative, acquired or learned, and still others are implicitly acquired through time spent in a particular environment (Stanovich, 2011).

Whatever the route, or combination of routes, the process by which biases become part of someone’s response repertoire appears to be robust and enduring. Consequently, we should expect that in order to effect CBM we need to recognise certain prerequisites: (Croskerry et al., 2017b)

1. Those engaged in CBM should be accomplished in detection and identification of JDM biases in themselves and others.
2. CBM is unlikely to work with a casual or limited intervention—it needs to be explicit, forceful and sustained.
3. It will likely require multiple interventions, and possibly forcing functions.
4. Given the multiple aetiologies of JDM biases we should expect that different CBM strategies will be required—it is unlikely one size will fit all.
5. We should anticipate that all CBM interventions will probably weaken over time and, therefore, will need lifelong maintenance.

The CBM climate is now looking more promising. In recent times, major behavioural changes have been accomplished that at one time might have seemed unthinkable: widespread cigarette smoking cessation, compliance with seat belts while driving, reduced instances of drunk driving, climate change awareness, increased organ donation, recycling, and many others. These behavioural changes have been accomplished through a repertoire of interventions ranging from forcing functions to simple nudging. Reviews of CBM strategies used in Medicine have shown a wide variety of strategies (Croskerry et al., 2017b), many of which appear effective (Ludolph & Schulz, 2017).

### Other factors that influence decision-making

In an ideal world, decision-makers would all be well-slept, well-rested, well-nourished, well-hydrated, in good humour, and have a not unreasonable work load. In the real world, however, homeostatic factors that form the C cluster in Fig. 1, may all interfere with clinical decision-making. The more obvious homeostatic violations, such as sleep deprivation, have a significant impact on diagnostic performance. In one study, diagnostic failure was increased fivefold in medical trainees following a level of sleep deprivation commonly experienced in many medical settings (Landrigan et al., 2004). The effects of major disruptions of the circadian rhythm are significant. A variety of neurocognitive deficits result from the sleep deprivation associated with night shift work in clinical settings (Croskerry et al., 2008b). However, even less draconian challenges may have a noticeable impact on clinical decision-making. A diurnal decay in the quality of decision-making was observed in a classic study of Israeli judge’s decisions (Danziger et al., 2011), an effect that has been replicated in a variety of medical settings during the normal work day (Chan et al., 2009; Dai et al., 2015; Hsiang et al., 2019; Kim et al., 2018; Linder et al., 2014; Philpot et al., 2018; Singh et al., 2016), i.e. fatigue effects occur as part of the normal diurnal day.

### Critical thinking

Despite some of the reservations expressed here, there are encouraging signs of a growing awareness of the importance of clinical decision-making and the role of critical thinking in medicine. The Foundation for Critical Thinking has defined its standards: clarity, accuracy, precision, relevance, depth, breadth, logic, significance, and fairness. Of themselves, they make sense, and few would argue that they are not appropriate for sound reasoning and decision-making. They are specified in plain language that is easily understood. Linda Elder, an Educational Psychologist and currently President of the Foundation for Critical Thinking notes that critical thinkers:

“…work diligently to develop the intellectual virtues of intellectual integrity, intellectual humility, intellectual civility, intellectual empathy, intellectual sense of justice and confidence in reason. They realise that no matter how skilled they are as thinkers, they can always improve their reasoning abilities and they will at times fall prey to mistakes in reasoning, human irrationality, prejudices, biases, distortions, uncritically accepted social rules and taboos, self-interest, and vested interest.” (Elder, 2007).

Again, these observations are easily understood and most would find them undisputable. However, the devil is in the details. Exactly how do we improve human reasoning, rationality and avoidance of bias? This, perhaps, is the greatest challenge. While the evidence for the efficacy of critical thinking interventions in education is good (Abrami et al., 2014; Higgins et al., 2005), specific efforts to introduce critical thinking into the medical undergraduate curriculum are rare. An exception is a study by Bonifacio et al. (2019) at the University of Pittsburgh School of Medicine. Third-year medical students exposed to a clinical reasoning intervention showed superior performance in tasks involving written reasoning skills and diagnostic reasoning and spent more time discussing clinical reasoning with their physician instructors.

### Certainty and uncertainty

An important feature of decision-making is the degree of confidence the decision-maker places in their decisions. Outside the medical context, a recognised bias towards overconfidence appears to be common. There is a general tendency for people to describe themselves as above average in a variety of skills, and a universal tendency for people to believe their judgements and beliefs are correct. Three biases, overconfidence, certainty, and optimism have been identified as underlying this tendency (Silveira & Rockman, 2021). In part, they appear to arise through a failure in metacognition or an inability by the decision-maker to step back and realistically judge their own competence. Those who overestimate their abilities are often the least skilled, referred to as the Dunning–Kruger effect.

This is compounded further in the course of professional training in medicine where trainees are encouraged to express confidence and certainty in their interactions with patients (Silveira & Rockman, 2021), perhaps because expressing confidence in the diagnosis and treatment is likely to make it more believable. As William James noted, ‘precursive faith,' that which gets ahead of the evidence, can bolster the cure. Equivocation and hesitancy do not work so well in Medicine, may be counter-therapeutic, and may result in therapeutic inertia.

There are additional problems with certainty bias. It may fuel other cognitive biases and exacerbate JDM errors (Silveira & Rockman, 2021). Further, as others have noted, knock-on effects of biases may occur (Dror et al., 2017), such that contributing to a certainty bias may pave the way for other biases to intrude, much like an anchoring and adjustment bias may lead to confirmation bias (Croskerry, 2000).

### Patient and clinician expectations

Throughout healthcare, there appear to be fundamental expectations from patients and physicians alike. Many patients appear willing to believe that clinicians have it within their power to competently diagnose disease, and usually effect a cure. In some ways, the inherent strength of this belief may be advantageous in that it may augment the placebo effect and accelerate the resolution of a disease. Given that illness itself is often a temporary state, and that the majority of illnesses will resolve of their own accord, it may not be such a bad thing. Whatever the perception of the patient, however, few are actually aware of the complexity of the diagnostic process or of the multiple factors that may influence it, including their own role. When they do develop significant symptoms, many come to expect that sophisticated and expensive technology and testing should be available for the asking, without regard to inherent costs and potential injury, e.g. a CT scan may be expected by the parents of a child with a mild head injury, without regard to the damaging effects of radiation and the potential for ‘false positive’ findings, benign radiologic discoveries that result in unnecessary treatment. Expensive screening tests need to be reconciled with lead time bias—in some cases, early detection of cancer may not actually change survival time, although there is a widespread popular belief that the sooner something is detected the better the outcome.

For their part, clinicians need to accept that not everything they can do for a patient should always be done. It is a time, too, of greater patient autonomy and patients taking critical decisions in their management, which may have significant repercussions for decisions physicians have to make. A refusal to be vaccinated can lead to resource management issues and in some cases death of the patient and others that might easily have been avoided if patients were coached on and heeded rational decision-making.

In their clinical decision-making, physicians may be obliged to the stewardship of resources not only because of their potentially harmful effects and the possibility of iatrogenic harm but also because they may be finite within healthcare systems; some level of judicious rationing may be required. More training should be directed towards the clinical JDM processes that promote choosing wisely. Finally, a crucial aspect of clinical JDM in real life is that it is not a simple academic exercise but may have significant morbidity and mortality associated with it.

### Interventions

Much of clinical judgement and decision-making depends upon core principles in cognitive science that have been developed over the last century or so, and which have become increasingly refined in the past 50 years. The argument has been made here that the general lack of training in cognitive science in the medical undergraduate curriculum ultimately diminishes the likelihood of raising the level of rational decision-making across the board in clinical medicine. Direct calls for the introduction of cognitive science into the medical curriculum have been made in the past (Croskerry, 2000; Croskerry & Nimmo, 2011; Elstein, 2009; Redelmeier et al., 2001; Royce et al., 2019) which might lead to an increased tendency to embrace cognitive solutions for problems in clinical decision-making.

Medicine has, at least, one notable success in curricula change. In the 1970s, statistical methods used in medical studies came under increasingly serious challenge. Not only were inappropriate methods being used but wrong conclusions were being drawn from the data. In one report, it was estimated that more than 50% of medical studies were statistically flawed. Not only were statisticians concerned but physicians, too, were increasingly worried about having to use statistical techniques they did not fully understand (Altman & Bland, 1991; Appleton, 1990). This led to a consensus among regulatory authorities that statistics should be an integral part of medical education, and in the UK, the General Medical Council made a recommendation in 1980 that statistics be included in the training of doctors. Going forward, all UK medical schools included training in statistics within the medical education syllabus. This example demonstrated, at least, that it was possible for the medical establishment to undergo a significant change in response to a specific need and raises optimism that a similar initiative, perhaps a more significant one, might be taken for the introduction of cognitive science into the medical undergraduate curriculum.

A further possibility might be an intervention at the candidate selection level. Medical school applicants are currently evaluated on a wide range of attributes and traits that commonly include measures of academic competence, traditionally in the pure sciences. More recently, good judgement and critical thinking are being recognised as desirable. Given that rationality is distributed much like intelligence, and can be measured, it would be possible to give some weight to candidates who have a high rationality quotient (RQ) (Stanovich et al., 2016). Thus, intellectual traits associated with rationality would already be present at the entry to medical school, and high RQ medical students might be expected to ultimately acquire higher levels of adaptive expertise in clinical decision-making.

## Conclusions

This overview has raised some key aspects of the relevance of cognitive science to clinical decision-making. The most critical decisions that clinicians make are mostly around diagnosis, a process that begins with pattern recognition and progresses through discrimination to matching with an established knowledge base. Through a cognitive autopsy approach, it appears that JDM failures arise mostly from cognitive processes that, at their most distal, appear to involve cognitive biases. Clinicians generally appear to have been reluctant to accept this view for a variety of reasons, in part due to a lack of awareness of the universality of JDM biases, and, in those that are aware, the notion that biases are difficult to mitigate. However, there appear to be increasing grounds for accepting that CBM can now be successfully accomplished. Explicit teaching about clinical decision-making in medical training should emphasise the critical role of cognitive science, as well as the unique role that medicine may play in its future development. Deliberate selection of candidates for medical school with high RQ scores might raise the level of rationality in clinical decision-making in medical practice.

## Data Availability

Not applicable. No study was done.
